# Outcomes of Aortic Valve Replacement Via Right Anterior Minithoracotomy and Central Cannulation *Versus* Conventional Aortic Valve Replacement in Obese Patients

**DOI:** 10.21470/1678-9741-2021-0098

**Published:** 2022

**Authors:** Burcin Abud, Onur Saydam, Aysen Yaprak Engin, Kemal Karaarslan, Ayse Gul Kunt, Mustafa Karacelik

**Affiliations:** 1 Cardiovascular Surgery Department, University of Health Sciences İzmir Tepecik Research and Education Hospital, Izmir, Turkey.; 2 Department of Pediatric Cardiac Surgery, University of Health Sciences Izmir Dr. Behcet Uz Children’s Hospital, Izmir, Turkey.

**Keywords:** Obesity, Minimally Invasive Surgical Procedures, Aortic Valve, Treatment Outcomes

## Abstract

**Introduction:**

The minimally invasive implantation of aortic valve prosthesis via thoracotomy has numerous advantages and is comfortable, especially during the early postoperative period. Disadvantages of this method include peripheral vessel complications and groin infections. Central cannulation (direct aortic cannulation with superior vena cava cannulation) eliminates these drawbacks. In this report, we evaluated this method of treatment in patients with obesity.

**Methods:**

We retrospectively analyzed the medical records of 21 obese patients with severe aortic stenosis who underwent minimally invasive aortic valve implantation via thoracotomy and central cannulation with a bovine pericardial aortic prosthesis between 2017 and 2021. We compared these records with the medical records of 27 obese patients with severe aortic stenosis who underwent conventional aortic valve surgery.

**Results:**

Mean cross-clamp and cardiopulmonary bypass times were similar in both groups. Operating time was significantly longer in the minimally invasive group (P <0.05). In the minimally invasive group, acute renal failure occurred in 2 patients. In terms of postoperative complications, deep sternal wound infection/sternal instability was much higher in the conventional group. This was not statistically significant (P=0.090). Minimally invasive operated patients had a comfortable early postoperative period, with a mean visual analog scale for pain of 1.10±0.83 (no pain-mild pain). When we assessed patient satisfaction with the postoperative period, 13 patients were extremely satisfied, 7 patients were satisfied, and 1 patient was quite satisfied.

**Conclusion:**

Minimally invasive aortic valve implantation via thoracotomy and central cannulation is a safe and effective treatment for obese patients.

**Table:** Abbreviations, Acronyms & Symbols

AVR	= Aortic valve replacement
BMI	= Body mass index
c-AVR	= Conventional aortic valve replacement
CCT	= Cross-clamp time
CPB	= Cardiopulmonary bypass
FS	= Full sternotomy
ICU	= Intensive care unit
Mini-AVR	= Minimally invasive aortic valve replacement
OT	= Operation time
RAMT	= Right anterior minithoracotomy
TAVI	= Transcatheter aortic valve implantation

## INTRODUCTION

Aortic valve replacement (AVR) via median full sternotomy (FS) is the conventional treatment for aortic valve disease (c-AVR). However, AVR has high morbidity and mortality rates, especially in the obese population^[1]^. Prolonged mechanical ventilation time, intensive care unit (ICU) and hospital stays, and risk of deep and superficial sternal wound infections and sternal dehiscence increase with the degree of obesity^[2,3]^. Alternative treatment methods with more reasonable mortality and morbidity rates have recently emerged, including minimally invasive aortic valve replacement (mini-AVR) via thoracotomy and transcatheter aortic valve implantation (TAVI)^[4,5]^. However, TAVI is not feasible in all patients, perioperative and postoperative complications are common, and long-term durability is controversial^[1]^. Although peripheral cannulation is more frequently performed in mini-AVR, central cannulation is an alternative^[6]^. The mini-AVR with peripheral cannulation has a lower risk and is more comfortable for patients, especially in the early postoperative period^[7,8]^. However, there remains a risk of peripheral vessel and groin complications, especially in obese patients^[9]^. Although exposure can be limited, central cannulation (direct aortic and superior vena cava cannulation) eliminates groin complications. In this report, we evaluate obese subjects who were treated with mini-AVR with central cannulation.

## METHODS

**Fig. 1:**
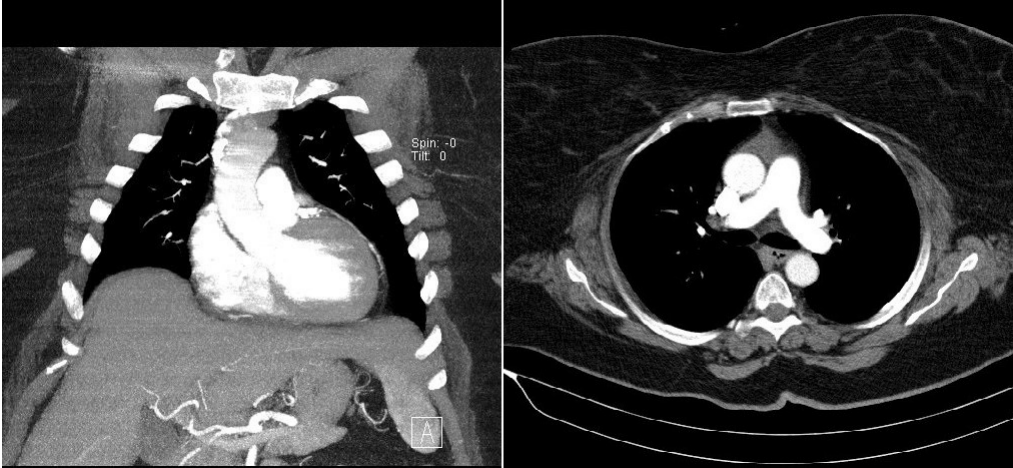
Preoperative computed tomography scan. Patients are suitable for this procedure if, at the level of the main pulmonary artery, the ascending aorta is rightward.

Twenty-one obese patients underwent mini-AVR via central cannulation between 2017 and 2021. All procedures were performed by two experienced heart valve surgeons. Sutureless bovine pericardial aortic prostheses (Perceval S, Liva Nova Sorin, Italy) or stented bovine pericardial aortic prostheses (Crown PRT, Liva Nova Sorin, Italy) were used. Patients’ age ranged from 58 to 82 years, with a mean age of 69.4±7 years. Obesity was defined as a body mass index (BMI) >30 kg/m^2^. Obese patients with isolated severe aortic valve stenosis or regurgitation were included. Exclusion criteria included a history of right pleurisy or pleural effusion with adhesion formation, chest wall deformities, previous major cardiac surgery involving pericardium opening, right-sided thoracic surgery, porcelain aorta, aneurysmal dilatation or ascending aortic dissection (≥4.5 cm) and concomitant significant coronary artery stenosis or other valve disease. The diagnosis of aortic valve disease was made by cardiologists with transthoracic echocardiography. Decisions for surgery were made at the Cardiology-Cardiovascular Surgery council. The American College of Cardiology and American Heart Association guideline published on 2014, titled “Guideline for the Management of Patients with Valvular Heart Disease”^[10]^, was followed. All patients underwent computed tomography scan during the preoperative period, and patients were considered for this procedure if the ascending aorta was rightward (more than half was located to the right of the right sternal border) at the level of the main pulmonary artery (Figure 1). All patients had severe aortic stenosis.

Preoperative demographic data were recorded after obtaining a medical history and performing the physical examination (Table 1). This study was approved by the ethics committee of the University of Health Sciences İzmir Tepecik Research and Education Hospital (No. 2020/14-16). The study complied with the Declaration of Helsinki. All patients provided written consent to use their data. Intraoperative data and postoperative outcomes were recorded.

**Table 1:** Patients’ preoperative demographics.

	mini-AVR (n=21)	c-AVR (n=27)	P
Age (years), mean±SD	69.43±7.11	71.22±6.14	0.235
F/M, n	16-mai.	17-out.	
BMI (kg/m^2^), mean±SD	36.39±5.21	36.18±4.65	0.983
Impaired renal function, n (%)	4 (19)	5 (18.5)	0.623
Peripheral artery disease, n (%)	1 (4.8)	4 (14.8)	0.262
Carotid artery disease, n (%)	4 (19)	4 (14.8)	0.495
Chronic lung disease, n (%)	9 (42.9)	11 (40.7)	0.883
Diabetes (IDDM), n (%)	12 (57.1)	13 (48.1)	0.536
Ejection fraction (IQR)	0.45 (0.30-0.55)	0.46 (0.30-0.60)	0.648
EuroSCORE II (%), mean±SD	3.76±2.15	3.28±2.17	0.554

AVR=aortic valve replacement; BMI=body mass index; EuroSCORE=European System for Cardiac Operative Risk Evaluation; F=female; IDDM=insulin-dependent diabetes mellitus; IQR=interquartile range; M=male; SD=standard deviation

Postoperative regional pain was measured by a visual analog scale for pain (0-10 rating, with 0 being no pain and 10 indicating the worst pain ever), and we assessed satisfaction about the postoperative period with a 5-point Likert scale (0 = extremely satisfied, 1 = satisfied, 2 = quite satisfied, 3 = dissatisfied, 4 = completely dissatisfied). We compared these records with the medical records of 27 obese patients (BMI >30 kg/m^2^) who underwent c-AVR by the same surgeons between 2014 and 2021. In these patients, a sutureless bovine pericardial aortic prosthesis (Perceval S, Liva Nova Sorin, Italy) or a stented bovine pericardial aortic prosthesis (Crown PRT, Liva Nova Sorin, Italy) was used.

### Surgical Procedure

All patients received the same medications, including propofol (2-3 mg/kg), fentanyl citrate (10-15 µg/kg), and pancuronium bromide (0.1 mg/kg). Following endotracheal intubation with a left-sided double-lumen tube, the anesthesia maintenance dose included intravenous propofol (2-5 mg/kg/h) and fentanyl citrate (5-10 µg/kg/h). Inhalational anesthesia was continued with sevoflurane. External defibrillator pads were placed on the chest wall.

**Fig. 2:**
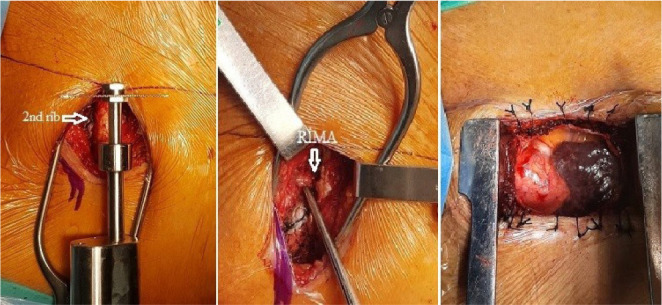
Exposure of the heart and its vessels.

**Fig. 3:**
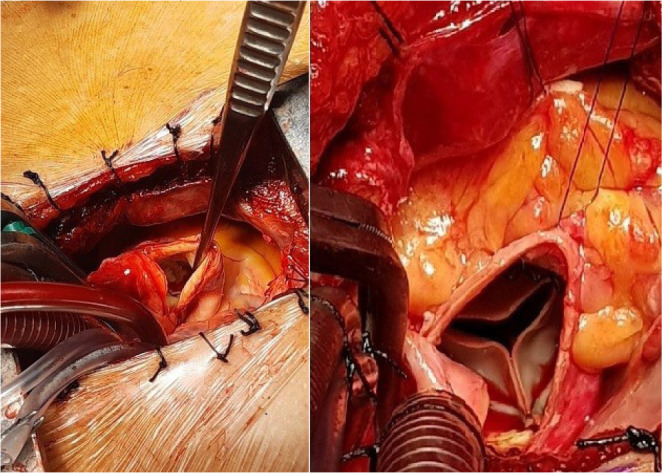
Aortotomy and implantation of the aortic prothesis.

For exposure, we incised through the right 2^nd^ intercostal space and transected the 2^nd^ rib (and the 3^rd^ rib if necessary). The right internal thoracic artery was ligated and divided. The pericardium was partially opened and fixed to the skin with suspension sutures (Figure 2). Aortic cannulation was performed on the anterior surface of the distal ascending aorta with a straight arterial cannula (Easyflow 23Fr, Liva Nova Sorin, Italy). Venous cannulation was conducted on the superior vena cava with an Optiflow venous return cannula (29Fr, Liva Nova Sorin, Italy). After cross-clamping and antegrade cold blood cardioplegia in the aortic root (and in the coronary ostia every 20 minutes), the aortic valve was implanted with venous vacuum-assisted cardiopulmonary bypass (with a negative pressure of approximately -40 to -60 mmHg) and venting of the right upper pulmonary vein. The aortic valve was implanted with a sutureless bovine pericardial aortic prosthesis (Perceval S, Liva Nova Sorin, Italy) in 17 patients and a stented bovine pericardial aortic prosthesis (Crown PRT, Liva Nova Sorin, Italy) in 4 patients (Figure 3). We used standard techniques for the sutureless and stented valve implantations. The transected rib was reattached to the sternum with a size “0” polyglactin suture. Intraoperative transesophageal echocardiography was used for heart deaeration and valve assessment. Drainage tubes were inserted into the mediastinum and the right pleural area. We opened the right pleural cavity so that the pericardial cavity seepage could drain into the pleural area. This approach does not require additional minimally invasive instruments, only standard equipment and instruments.

### Statistical Analysis

All data were analyzed using the Statistical Package for the Social Sciences 16.0 for Windows (SPSS Inc., Chicago, Illinois, USA). Continuous variables, except for the duration of mechanical ventilation and ICU and hospital stays, were reported as mean±standard deviation or median with interquartile range. Duration of mechanical ventilation and length of stay in ICU and in the hospital were reported as medians with minimum (min) and maximum (max) values. Categorical variables were expressed as a percentage. Continuous variables were compared using the Mann-Whitney U-test and categorical variables were compared with the chi-square test or Fisher’s exact test. A *P* <0.05 was considered to be a statistically significant indicator of differences.

## RESULTS

The mean age of the patients was 69.4±7 years (range, 58-82), and the mean BMI was 36.4±5.2 (range, 30.1-48.2) in the mini-AVR group. Five patients were morbidly obese, with BMIs of 40.5, 42.2, 42.6, 45.8 and 48.2, respectively. In the c-AVR group, the mean age of the patients was 71.2±6 years (range, 60-80), and the mean BMI was 36.2±4.7 (range, 30.4-45.9). In this group, 4 patients were morbidly obese, with BMIs of 43.3, 43.5, 44.9 and 45.9, respectively. There was no significant difference between both groups in terms of demographic characteristics, BMI and operative risk, calculated with EuroSCORE II (Table 1).

In the mini-AVR group, a sutureless bovine pericardial aortic prosthesis was used in 17 patients and a stented bovine pericardial aortic prosthesis was used in 4 patients. Ten patients in the sutureless group had a Medium-M size (22-23 mm) implanted valve, while 7 patients had a Large-L size (24-25 mm) valve. The 4 patients in the stented group had a 23 mm valve. In the c-AVR group, 22 patients had a sutureless bovine pericardial aortic valve implanted and 5 had a stented bovine pericardial valve implanted. In both groups, 19% of the patients had a stented valve implanted. The mean cross-clamp (CCT) and cardiopulmonary bypass (CPB) times were similar in both groups. Operation time (OT) was significantly longer in the mini-AVR group (*P*=0.000) (Table 2). Intraoperative transesophageal echocardiography showed successful positioning of the prosthesis in all patients, and no deaths occurred during the operation.

**Table 2:** Patients’ intraoperative characteristics.

	mini-AVR (n=21)	c-AVR (n=27)	P
Cross-clamp time (min), mean±SD	50.43±11.64	48.93±12.23	0.617
CPB time (min), mean±SD	75.10±11.72	74.44±12.79	0.851
Operation time (min), mean±SD	168.19±12.47	135.30±12.36	0.000
Conversion rate, n (%)	0 (0.0)	-	
Prosthesis size (n), sutureless			
S (19-21 mm; mean size 20 mm)	0	0	
M (22-23 mm; mean size 22.5 mm)	10	13	
L (24-25 mm; mean size 24.5 mm)	7	8	
XL (26-27 mm; mean size 26.5 mm)	0	1	
Prosthesis size (n), stented			
19 mm	0	0	
21 mm	0	0	
23 mm	4	4	
25 mm	0	1	
Diameter of implanted prosthesis (mm), mean±SD	23.26±0.92	23.41±1.13	0.796

CPB=cardiopulmonary bypass; SD=standard deviation

Postoperative use of packed red blood cells was significant lower in the mini-AVR group (*P*=0.002). A mean of 0.90±0.83 units of packed red blood cells was used, and a mean of 0,71±0.72 units of fresh frozen plasma was used in the mini-AVR group. Two patients experienced acute renal failure on the 1^st^ postoperative day, but they recovered quickly. Three patients had new-onset postoperative atrial fibrillation, which improved with medication. No postoperative complications occurred in the other patients. In the c-AVR group, acute renal failure occurred in 3 patients, new-onset atrial fibrillation occurred in 3 patients and superficial wound infection occurred in 1 patient. Four patients had deep sternal wound infection with instability. When we evaluated the postoperative complications of both groups, deep sternal wound infection with instability was much higher in the c-AVR group. However, this was not statistically significant (*P*=0.090).

The mean mechanical ventilation time was 6 hours (min 4 h; max 12 h), the mean ICU stay was 2 days (min 1 day; max 7 days), and the mean hospital stay was 7 days (min 5 days; max 14 days) in the mini-AVR group. Mean mechanical ventilation time and mean ICU stay were statistically significantly reduced in comparison to c-AVR patients (*P*=0.001; *P*=0.006).

The minimally invasive operated patients had a comfortable early postoperative period, with a mean visual analog scale for pain of 1.10±0.83 [“0” (no pain) was reported in 5 patients, “1” in 10 patients, “2” in 5 patients, and “3” in 1 patient.] Thirteen patients were extremely satisfied with the postoperative period, 7 were satisfied, and 1 was quite satisfied (Table 3).

**Table 3:** Patients’ postoperative outcomes.

	mini-AVR (n=21)	c-AVR (n=27)	P
Re-exploration for major bleeding, n (%)	0 (0.0)	1 (3.7)	0.563
Cardiac arrest, n (%)	0 (0.0)	0 (0.0)	
Myocardial infarction, n (%)	0 (0.0)	0 (0.0)	
New-onset atrial fibrillation, n (%)	3 (14.3)	3 (11.1)	0.537
Need for permanent pacemaker, n (%)	0 (0.0)	0 (0.0)	
Stroke, n (%)	0 (0.0)	0 (0.0)	
Renal failure, n (%)	2 (9.5)	3 (11.1)	0.621
Deep sternal wound infection or dehiscence, n (%)	0 (0.0)	4 (14.8)	0.090
Superficial wound infection, n (%)	0 (0.0)	1 (3.7)	0.563
In-hospital mortality, n (%)	0 (0.0)	0 (0.0)	
Packed red blood cells (units), mean±SD	0.90±0.83	2.07±1.44	0.002
Fresh frozen plasma (units), mean±SD	0.71±0.72	1.15±0.99	0.125
Mechanical ventilation time (hours)	6 hours (min 4; max 12)	8 hours (min 5; max 14)	0.001
ICU stay (days)	2 days (min 1; max 7)	3 days (min 2; max 9)	0.006
Hospital stay (days)	7 days (min 5; max 14)	9 days (min 5; max 20)	0.395
Patient pain/satisfaction data			0.000
Postoperative regional pain, mean±SD	1.10±0.83	5.67±2.11	
Postoperative satisfaction, mean±SD	0.43±0.6		
6-month follow-up			
Mean gradient (mmHg)	9.1±3.0	8.7±3.0	0.690
Peak gradient (mmHg)	19.3±4.2	19.1±4.1	0.834

ICU=intensive care unit; SD=standard deviation

At the 6-month follow-up, echocardiographic controls showed a normally functioning aortic valve with no migration or dislodgement and with low transvalvular pressure gradients in both groups (Table 3).

## DISCUSSION

AVR is the gold standard of treatment in patients with severe aortic valve stenosis, with a mortality rate of 0.5-2.6%^[11,12]^. With increasing life expectancy, severe aortic stenosis is frequently seen in elderly patients. In this patient population, AVR carries high morbidity and mortality rates, especially in people with significant comorbidities^[1,13]^. AVR is particularly risky in patients with a high BMI (>30 kg/m^2^). FS alone causes complications and carries high morbidity in obese patients. Mechanical ventilation time and ICU and hospital stays are longer in obese patients with FS. Additionally, sternal and superficial wound infections and sternal dehiscence are much more common in these patients^[2,3]^. To reduce the risks of sternotomy, several minimally invasive approaches have been developed, including partial sternotomy (upper or lower hemi-sternotomy with “T”, “inverted T”, “J”, “L”, or “V-shaped” incision), transverse sternotomy, and right anterior minithoracotomy (RAMT)^[9,12,14]^.

Partial sternotomy is the most widely used minimally invasive AVR procedure and has many benefits for elderly and obese patients. ElBardissi et al.^[15]^ confirmed the benefits of the minimally invasive approach in elderly, high-risk patients with aortic valve disease requiring AVR. Operative mortality was only 3% in these patients, and perioperative morbidity was low. Welp et al.^[14]^ compared the results of aortic valve surgery in obese patients with full and partial sternotomy. They reported reduced need for blood transfusion and mechanical ventilation times and shorter ICU length of stay in the partial sternotomy group.

The second most frequently used approach in minimally invasive AVR is the RAMT approach. Few studies have evaluated the outcomes of aortic valve implantation via RAMT in elderly and obese patients. Gilmanov et al.^[8]^ compared the outcomes of elderly patients who have undergone aortic valve implantation via FS and RAMT. They reported similar morbidity and mortality rates in both groups. The RAMT group had a faster recovery and reduced length of hospital stay. Similar results were reported by Sharony et al.^[16]^ and Santana et al.^[17]^, who evaluated obese patients (BMI >30 kg/m^2^) and reported lower morbidity and mortality rates in the RAMT group as compared with the FS group.

In studies that directly compared partial sternotomy and RAMT, minimally invasive AVR using RAMT was associated with better outcomes. Postoperative atrial fibrillation and transfusion rates were lower, and duration of mechanical ventilation and ICU and hospital stays were shorter in the RAMT group, despite longer cross-clamp and CPB times^[18]^.

Our study population includes obese individuals. Compared to our conventionally operated patients, the mean CCT and CPB times were similar, only the mean OT was significantly longer in the mini-AVR group. This is because it takes longer to open the surgical field and expose the heart and its vessels. Due to aneurysmal dilatation of the ascending aorta (≥4 cm), in 4 patients of the mini-AVR group and in 5 patients of the c-AVR group, a stented aortic valve was used (19% of the patients in each group). Mean CCT, CPB and operation times were about 20 minutes shorter with sutureless prostheses as compared with stented prostheses in both groups. No complications or deaths occurred during the operation in both groups, and none of the minimally invasive procedures were converted to FS.

Previous studies reported that a minimally invasive approach for AVR does not increase the risk of patient-prosthesis mismatch^[14,19]^. In our study, the implanted aortic valve size was similar in both groups.

In the present study, the use of packed red blood cells was reduced by half in comparison to our conventional operated aortic valve implantations (*P*=0.002). Similar results were found by Welp et al.^[14]^ and Santana et al.^[17]^. We think that reduced use of packed red blood cells is associated with reduced surgical trauma with the minimally invasive approach.

In the mini-AVR group, the mean mechanical ventilation times were significant lower than those with conventional surgery. Similar reduced results were found by Welp et al.^[14]^ and Santana et al.^[17]^.

Sternotomy carries a high morbidity rate in obese patients. The risk of deep and superficial sternal wound infections and dehiscence increases with increasing degree of obesity^[2,3]^. Santana et al.^[17]^ reported reduced composite complications in the minimally invasive AVR group in comparison with the conventional AVR group, whereas complication rates were similar in the research by Welp et al.^[14]^. In our study, in the mini-AVR group the most common postoperative complication was atrial fibrillation. No deep or superficial sternal wound infection occurred in any patients. In the c-AVR group, deep sternal wound infection with sternal instability was the most common postoperative complication. Superficial wound infection occurred in 1 patient and new-onset atrial fibrillation in 3 patients. In terms of postoperative complications, deep sternal wound infection with sternal instability was very higher in the c-AVR group. However, this was not statistically significant (*P*=0.090). New-onset atrial fibrillation was the most postoperative complication in all 48 patients. Acharya et al.^[19]^ reported a positive correlation between BMI and new-onset atrial fibrillation.

Another disadvantage of this minimally invasive approach is the use of femoral cannulation and perfusion, which may lead to peripheral vessel complications and groin infections. Several studies have reported the drawbacks of femoral cannulation in minimally invasive AVR^[9,20]^. We used direct cannulation of the aorta and superior vena cava in a group of patients likely to experience postoperative groin complications. Therefore, peripheral vessel complications and groin infections were not observed in our patients.

Minimally invasive approaches are generally not preferred in obese patients because of insufficient surgeon experience and inadequate surgical field exposure. We found that our RAMT approach provides good operating field exposure. The suspended pericardium prevents the lung from entering the operating area and exposes the ascending aorta, the superior vena cava, the right ventricular outflow tract, the pulmonary artery, the upper right pulmonary vein, and part of the right atrium^[8]^. In 4 patients, the upper right pulmonary vein was not visible, and we had to transect the 3^rd^ rib. We do not use the venous cannula for the right atrium, so that it is not an obstacle in the operating field. Venous cannulation was performed in the superior vena cava only for better exposure. Vacuum-assisted venous drainage is effective in patients with a high BMI. Our minimally invasive approach requires no additional surgical training and can be performed with standard surgical instruments and equipment.

Our study evaluated the outcomes of RAMT aortic valve surgery in obese patients. Our findings make important contributions to the existing literature. Our conclusions are in line with previous studies and meta-analyses, which predominantly reveal better results for mechanical ventilation time, ICU length of stay, need for packed red blood cell transfusion, sternal infections/dehiscence, and postoperative comfort for minimally invasive AVR in obese patients.

## CONCLUSION

Minimally invasive AVR via thoracotomy and central cannulation, which reduces surgical trauma and protects peripheral vessels, is a safe and effective treatment option for obese patients. It is associated with low morbidity and mortality rates and enhanced comfort during the early postoperative period. Significant benefits in terms of reduced need for transfusion, shorter postoperative mechanical ventilation times and shorter ICU stays were found. We observed clear benefits in a group of patients who are likely to have increased postoperative morbidity and mortality.

### Limitations

Our retrospective, observational cohort study with prospective data collection carries all the limitations of a retrospective study.
